# *Klebsiella pneumoniae* Planktonic and Biofilm Reduction by Different Plant Extracts:* In Vitro* Study

**DOI:** 10.1155/2016/3521413

**Published:** 2016-11-28

**Authors:** Lucas De Paula Ramos, Carlos Eduardo da Rocha Santos, Daphne Camargo Reis Mello, Lígia Nishiama Theodoro, Felipe Eduardo De Oliveira, Graziella N. Back Brito, Juliana Campos Junqueira, Antonio Olavo Cardoso Jorge, Luciane Dias de Oliveira

**Affiliations:** Department of Biosciences and Oral Diagnosis, São Paulo State University (Unesp), Institute of Science and Technology, São José dos Campos, Francisco José Longo 777, São Dimas, 12245-000 São José dos Campos, SP, Brazil

## Abstract

This study evaluated the action of* Pfaffia paniculata* K.,* Juglans regia* L., and* Rosmarius officinalis* L. extracts against planktonic form and biofilm of* Klebsiella pneumoniae* (ATCC 4352). Minimum inhibitory concentration (MIC) and minimum microbicidal concentration (MMC) values were determined for each extract by microdilution broth method, according to Clinical and Laboratory Standards Institute. Next, antimicrobial activity of the extracts on biofilm was analyzed. For this, standardized suspension at 10^7^ UFC/mL of* K. pneumoniae* was distributed into 96-well microplates (*n* = 10) and after 48 h at 37°C and biofilm was subjected to treatment for 5 min with the extracts at a concentration of 200 mg/mL. ANOVA and Tukey tests (5%) were used to verify statistical significant reduction (*p* < 0.05) of planktonic form and biofilm*. P paniculata *K.,* R. officinalis* L., and* J. regia* L. showed reductions in biomass of 55.6, 58.1, and 18.65% and cell viability reduction of 72.4, 65.1, and 31.5%, respectively. The reduction obtained with* P. paniculata *and* R. officinalis* extracts was similar to the reduction obtained with chlorhexidine digluconate 2%. In conclusion, all extracts have microbicidal action on the planktonic form but only* P. paniculata *K. and* R. officinalis* L. were effective against biofilm.

## 1. Introduction

*Klebsiella pneumoniae* belongs to* Enterobacteriaceae* family and it has emerged as an important pathogen, responsible for nosocomial infections, focusing on urinary (UTIs) and respiratory tracts and blood infections. Although it is a common colonizer of skin, gastrointestinal tract, and nasopharynx, it is a risk for patients hospitalized for long periods and with compromised immune system. It is also responsible for more than 70% of infections in humans. When organized in biofilms, virulence factor that makes it an extremely resistant microorganism, it offers great resistance to the diffusion of antimicrobial drugs [1–4], with little or no treatment option being developed [5].

Due to the increasing resistance to antimicrobial drugs, especially after the 70s, when several strains emerged with a high antimicrobial resistance rate, such as cephalosporins and fluoroquinolones, new treatment options became extremely important. Thus, studies with alternative methods, such as herbal medicines, are growing as a promising field to combat resistant strains. A great number of studies tried to explain the action potential of natural extracts; however, nowadays, there are over 3000 species that have not been evaluated. Therefore, the extracts selected for this study show little or no information regarding their antimicrobial potential [6–8].

*Pfaffia paniculata* K. is a common root in South America, especially in Brazil, popularly known as Brazilian ginseng. It belongs to* Amaranthaceae* family and it is known for its anti-inflammatory potential. Studies indicate that its action potential can be much higher, even against tumor cells in different sites. However, regarding its antimicrobial activity, there is nothing so far described in the literature [9, 10].

*Juglans regia* L., which belongs to* Juglandaceae* family, popularly known as walnut, is a common tree in South Eastern Europe. Its fruits have high nutritional content and are rich in antioxidants. Researches around the walnut show anti-inflammatory, analgesic, and antimicrobial actions. Its antimicrobial activity was tested against Gram-positive and Gram-negative microorganisms and shows promising results, varying according to the type of extract [11, 12].

*Rosmarinus officinalis* L. is a shrub from the mediterranean region, drift of* Lamiaceae *family that has been used for medicinal purposes in the treatment of bacterial and fungal infections [13]. It presents preventive and/or curative action of diseases such as asthma, tonsillitis, nasal obstruction, and constipation. It has monoterpenes with antibacterial and antifungal potential, especially against Gram-positive bacteria [14].

Taking into consideration what has been exposed before, this study aimed to evaluate the antimicrobial action of glycolic extracts of* P. paniculata *K,* J. regia* L., and* R. officinalis* L. against* K. pneumoniae* planktonic suspension and biofilm.

## 2. Materials and Methods

Glycolic extracts of* Pfaffia paniculata*,* Juglans regia,* and* Rosmarinus officinalis* were purchased from Mapric company with the appropriate reports and specifications.

*Klebsiella pneumoniae* ATCC 4352, from Microbiology and Immunology Laboratory–Institute of Science and Technology/UNESP, was used for the evaluation of antimicrobial activity of the glycolic extracts.

### 2.1. Minimum Inhibitory Concentration (MIC) and Minimum Microbicidal Concentration (MMC)

In order to determine MIC, microdilution broth method was used, according to the Clinical and Laboratory Standards Institute (CLSI), M7-A6 standard [15].

Inocula were prepared from a culture seeded on BHI agar (Brain Herth Infusion), incubated in bacteriological incubator at 37°C during 24 h in sterile physiological solution (0,9% NaCl) and standardized at spectrophotometer in 10^6^ cells/mL, according to the recommendations of NCCLS [15].

The test was performed in 96-well microplates (TPP, Zollstrasse, Switzerland), where 100 *μ*L of Mueller Hinton (Himedia, Mumbai, India) were added and 100 *μ*L of the extract only in the first well. From the first well, 10 serial dilutions were done. Next, 100 *μ*L of microbial suspension was added into each well and the plate was incubated for 24 h. With this method of serial dilution, concentration of each extract tested ranged from 100 to 0,19 mg/mL. MIC was found in the last well of the microplate with no turbidity.

MMC of the extracts was determined by inoculating 10 *μ*L of MIC, a higher and a lower concentration into Brain Heart Infusion agar (BHI, Himedia, Mumbai, India) and the drop technique was used for seeding. After 48 h at 37°C, the lowest seeded concentration that did not show growth on solid medium was determined as MMC.

### 2.2. Evaluation of the Antimicrobial Activity of the Extracts in Biofilms [16, 17]

The action of the extracts against* Klebsiella pneumoniae* monospecies biofilm was evaluated. The biofilms were formed in the bottom of 96-well plates. First,* K. pneumoniae* was suspended in Brain Hearth Infusion Broth (Himedia) and incubated at 37°C during 24 h. Next, the inoculum was washed twice with sterile saline (0.9% NaCl) and standardized suspensions containing 10^7^ UFC/mL were obtained with the aid of spectrophotometer (B582, Micronal, São Paulo, Brazil). A hundred microliters of microbial suspension were placed per well in each group (*n* = 10) in 96-well microplates. The plates were incubated for 90 min in bacterial incubator at 37°C under agitation of 75 rpm for initial adhesion. The plates were washed twice with sterile saline and 100 *μ*L/well of broth was added. Then the plates were incubated under the same conditions of initial adhesion for 48 h, with replacement of broth after 24 h.

#### 2.2.1. Treatment

After,* K. pneumoniae* biofilm was put in contact with each extract at the concentration of 200 mg/mL, separately, during 5 minutes. As a positive control, it was used 2% chlorhexidine digluconate and as a negative control 0.9% NaCl sterile. Subsequently, the extracts were removed and the biofilm was washed with sterile 0.9% NaCl.

Three independent experiments were performed, with 10 repetitions each, total *n* = 30 for each group.

#### 2.2.2. Biofilm Measuring

*Violet Crystal*. After exposure of biofilm to the extracts, 200 *μ*L of methanol was added in each well for 15 min. The liquid was removed and the plate was incubated for 24 h. After the incubation period, 200 *μ*L of crystal violet 1% (v/v) was added for 5 min. The wells were washed twice with 200 *μ*L of saline and acetic acid 33% (v/v). The absorbance of the plates was measured in a microplate reader at a wavelength of 570 nm. Optical density obtained was converted to reduction percentage of biofilms.

*Cellular Viability*. After exposure of biofilm to the extracts, 100 *μ*L/well of MTT solution (Sigma-Aldrich Co., Germany) was added and the plates were incubated, protected from light, during 1 h at 37°C. Plates were washed with sterile phosphate buffered saline (PBS) (Cultilab, Brazil) and 100 *μ*L/well of dimethyl sulfoxide (DMSO, Sigma) was added. Then, the plates were incubated again, protected from light, for 10 min at 37°C, under agitation for more than 10 min. Optical densities of the wells were measured by microplate reader at a wavelength of 570 nm, and the values obtained were converted to percentage of cell viability.

### 2.3. Statistical Analysis

The results were analyzed by ANOVA and Tukey test (*p* < 0.05) with Graphpad Prism 5.0 software.

## 3. Results

The results obtained with the broth microdilution test are shown in Table 1.

*P. paniculata* and* R. officinalis* showed MIC of 12.5 mg/mL and MMC of 25 mg/mL.* J. regia* showed MIC and MMC at the same concentration of 6.25 mg/mL.

**Figure 1:**
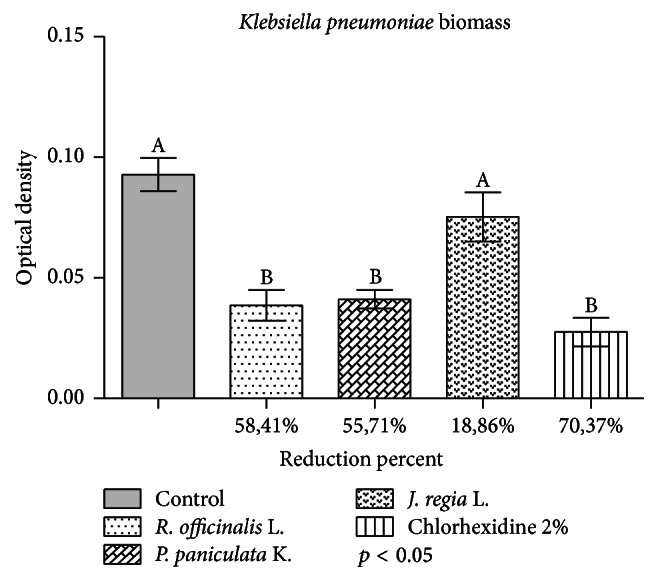
Application of glycolic extracts of* R. officinalis, P paniculata,* and* J. regia* at concentration of 200 mg/mL in* K. pneumoniae* biofilm during 5 minutes. Control group was exposed to 0.9% NaCl during 5 min. The results indicate the mean values in reduction of biomass in percentage compared to the control group of each extract. Letters (A or B) express the comparison of groups in statistical analysis (*p* < 0.05).

Results obtained with the application of the extracts in the biofilm in the crystal violet assay are shown in Figure 1.

*P. paniculata *and* R. officinalis* extracts showed statistically significant reduction when compared to the control group (*p* < 0.05). The highest mean percentages of reduction in the biofilm were 58.41% for* R. officinalis* and 55.71% for* P. paniculata*, statistically similar to 2% chlorhexidine digluconate, with reduction of 70.37%.

Although* J. regia* extract has shown a mean reduction of 18.86%, this was not statistically significant when compared to the control group (*p* > 0.05).

**Figure 2:**
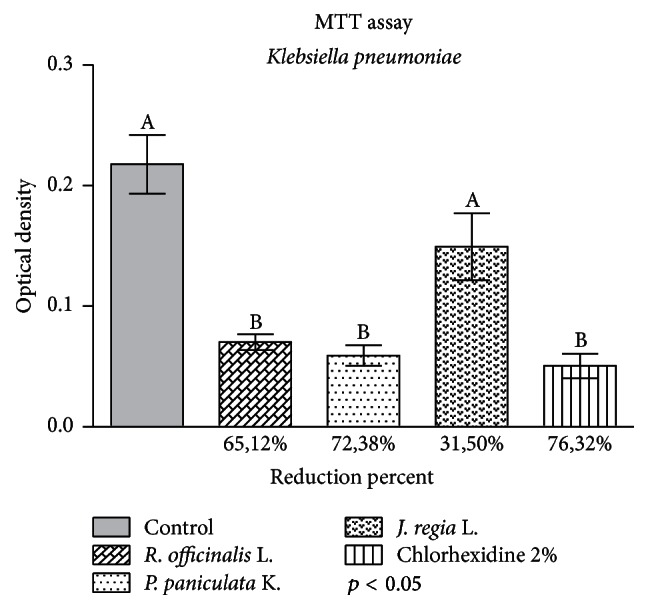
Application of glycolic extracts of* R. officinalis, P paniculata,* and* J. regia* at concentration of 200 mg/mL against* K. pneumoniae* biofilm during 5 min. Control group received 0.9% NaCl during 5 min. The results indicate the mean values of cell viability reduction in percentage of each extract compared to the control group. Letters (A or B) express the comparison of groups in statistical analysis (*p* < 0.05).

The results of cell viability test are in Figure 2.

The extracts of* R. officinalis* and* P. paniculata *showed statistically significant reduction when compared to the control group (*p* < 0.05). The mean reduction percentages in the biofilm were 72.38% for* P. paniculata *and 65.12% for* R. officinalis*, both statistically similar to 2% chlorhexidine digluconate, with reduction of 76.32%, and 31.5% for* J. regia*, statistically similar to the control group.

## 4. Discussion

The results of this study demonstrated that the natural extracts have antimicrobial activity against* K. pneumoniae*, a pathogen that offers great resistance to different antibiotics [1–4]. It is remarkable that this is a pioneering study, since it checks the antimicrobial action of* P. paniculata*,* J. regia*, and* R. officinalis* glycolic extracts with lack of information in the literature [6–8].

Microbial resistance is a growing threat to global public health [18]. In the last decade, emergence of resistance to antimicrobial drugs partially reversed the advances of antibiotics that occurred during the last 80 years (National Action Plan). The high incidence of infections caused by multiresistant bacteria makes the reevaluation of treatment regimens, with alternative options, a real need for health research [19].

Pleşca et al., in a retrospective study with 178 patients with severe sepsis associated with immunosuppression frames, pointed* K. pneumoniae* as the second most common infectious agent, behind* Escherichia coli*. Marwa et al., in a cross-sectional study with patients with and without HIV, found similar results to Pleşca et al. From clinical isolates, resistant to cotrimoxazole, a prophylactic antibiotic worldwide used against opportunistic infections, in patients with HIV or AIDS,* K. pneumoniae* appears to be the second most prevalent microorganism, behind only* E. coli.* These data only corroborate the importance of the results found in our study, especially regarding* R. officinalis* and* P. paniculata *extracts, that were able to significantly reduce biofilm [20, 21].

Our study has similar results to those found in the literature, according to plant or root species and type of extract tested, such as Kozłowska et al., who tested the aqueous and ethanol extracts of* R. officinalis* on standard strains of* K. pneumoniae* and they both showed inhibition on the strain ATCC 13883, but there was no inhibition on the strain ATCC 700603. In another research, Van Vuuren et al. tested different essential oils in combination with ciprofloxacin against different microorganisms and the best results were with* R. officinalis* essential oil associated with ciprofloxacin against* K. pneumoniae* NCTC 9633 [22, 23].

Pereira et al. used the aqueous extract of* J. regia* and found inhibition of* K. pneumoniae*. Abidi et al. demonstrated synergistic action of different extracts associated with antibiotics against clinical strains of* Staphylococcus epidermidis*, wherein the aqueous extract of* J. regia* was tested with several standard antibiotics such as gentamicin and vancomycin, among which cephalexin and erythromycin showed the best results. Farooqui et al. tested aqueous and methanolic extracts of* J. regia* on clinical and standard (ATCC) strains. Clinical strains of Gram-positive pathogens, such as* Staphylococcus aureus*, showed higher sensitivity to the extract; however, when tested on Gram-negative strains, the aqueous and methanolic extracts showed no significant antimicrobial activity, as well as the results obtained in our study. It is suggested that the antimicrobial action of* J. regia* against Gram-negative microorganisms must be strain dependent, showing greater effect on Gram-positive microorganisms. Thus, more research on Gram-positive microorganisms with different* J. regia* extracts could bring better results [24–26].

The results obtained with* P. paniculata* are difficult to discuss, since the articles in the literature address only their anticancer and antitumor [27, 28], anti-inflammatory [29], and cell activity modulator [30] effects, among others. However, until the present moment, there is no research with this extract regarding its antimicrobial potential, which makes our study of great importance, while opening new perspectives for studies with promising results.

Studies show that plants contain different types of monoterpenes in their composition.* P. paniculata *has a triterpene called pfameric acid and* J. regia* and* R. officinalis* have several types of monoterpenes hydrocarbons. Pham et al. reported that the monoterpene molecule has the ability to change the fluidity and the carrying of substances through the lipid bilayer. Thus, this change in the membrane permeability may have caused metabolism collapse and death of the microorganism [31–34]. The difference in susceptibility of the tested microorganism can be attributed to the rate at which the active components of the plant extracts seem to diffuse through the cell and into the phospholipids of cell membrane [35].

## 5. Conclusion

It was concluded that all glycolic extracts showed antimicrobial action against the planktonic form of* K. pneumoniae* but only* R. officinalis* and* P. paniculata* extracts, at 200 mg/mL, showed antimicrobial action against the biofilm. These results are promising and open new avenues for research with alternative antimicrobial methods.

## Figures and Tables

**Table 1:** Broth microdilution test results with concentrations corresponding to MIC and MMC for each extract.

	*Klebsiella pneumoniae*	
	MIC (mg/mL)	MMC (mg/mL)
*Pfaffia paniculata *	12,5	25
*Juglans regia*	6,25	6,25
*Rosmarinus officinalis *	12,5	25
